# Deciphering the heterogeneity dominated by tumor-associated macrophages for survival prognostication and prediction of immunotherapy response in lung adenocarcinoma

**DOI:** 10.1038/s41598-024-60132-4

**Published:** 2024-04-23

**Authors:** Jiazheng Sun, Hehua Guo, Yalan Nie, Sirui Zhou, Yulan Zeng, Yalu Sun

**Affiliations:** 1 Liyuan Hospital, Tongji Medical College, Huazhong University of Science and Technology, Wuhan, China; 2 Affiliated Hospital of Jining Medical University, Jining, China

**Keywords:** Lung adenocarcinoma, Tumor-associated macrophages, Prognostic signature, Immunotherapy, Tumor microenvironment, Cancer, Computational biology and bioinformatics, Biomarkers

## Abstract

Tumor-associated macrophages (TAMs) are a specific subset of macrophages that reside inside the tumor microenvironment. The dynamic interplay between TAMs and tumor cells plays a crucial role in the treatment response and prognosis of lung adenocarcinoma (LUAD). The study aimed to examine the association between TAMs and LUAD to advance the development of targeted strategies and immunotherapeutic approaches for treating this type of lung cancer. The study employed single-cell mRNA sequencing data to characterize the immune cell composition of LUAD and delineate distinct subpopulations of TAMs. The “BayesPrism” and “Seurat” R packages were employed to examine the association between these subgroups and immunotherapy and clinical features to identify novel immunotherapy biomarkers. Furthermore, a predictive signature was generated to forecast patient prognosis by examining the gene expression profile of immunotherapy-associated TAMs subsets and using 104 machine-learning techniques. A comprehensive investigation has shown the existence of a hitherto unidentified subgroup of TAMs known as RGS1 + TAMs, which has been found to have a strong correlation with the efficacy of immunotherapy and the occurrence of tumor metastasis in LUAD patients. CD83 was identified CD83 as a distinct biomarker for the expression of RGS1 + TAMs, showcasing its potential utility as an indicator for immunotherapeutic interventions. Furthermore, the prognostic capacity of the RTMscore signature, encompassing three specific mRNA (NR4A2, MMP14, and NPC2), demonstrated enhanced robustness when contrasted against the comprehensive collection of 104 features outlined in the published study. CD83 has potential as an immunotherapeutic biomarker. Meanwhile, The RTMscore signature established in the present study might be beneficial for survival prognostication.

## Introduction

Lung cancer ranks among the leading causes of mortality globally about cancer. The prevalence of lung adenocarcinoma (LUAD) has steadily risen, solidifying its status as one of the predominant subtypes of lung cancer in the current era. The utilization of immune checkpoint inhibitors (ICIs) in cancer therapy has recently witnessed a significant rise. The utilization of PD-1 inhibitors, combined with chemotherapy, has been employed to manage advanced or metastatic LUAD. The primary mode of action of PD-1 inhibitors is disrupting the binding between PD-1 and its corresponding ligand, PD-L1, thereby enabling the patient's immune system to selectively attack malignant cells^1–3^. The efficacy of CTLA-4 and PD-1 inhibitors has been investigated in clinical trials as a potential therapeutic approach for lung cancer^4^. Nevertheless, as a result of the inherent unpredictability associated with immune escape mechanisms and the complex tumor microenvironment (TME), it is important to note that only a specific subgroup of patients has the potential to achieve a curative response. On the contrary, some individuals may have acquired unforeseen resistance to ICIs^5^.

While previous studies have suggested that some indicators, such as tumor PD-L1 expression level^6^ and tumor mutational burden^7^, could potentially serve as predictors for the response to immunotherapy in LUAD^8^, the practical applicability of these predictions has not been consistently reliable. Instances of hyperprogressive disease after atezolizumab therapy have been documented, hence emphasizing the intricate and dynamic cellular interactions within the TME^9,10^.

The TME has diverse components, such as tumor cells, immune cells, blood arteries, fibrous tissue, and other elements^11^. The efficacy of immunotherapy was greatly influenced by the interplay between these components. A more detailed characterization of the various cell types inside the TME and their interactions will enable researchers to gain a deeper understanding of the immunological status of tumors and facilitate the identification of novel biomarkers.

Tumor-associated macrophages (TAMs) have been found to play a pivotal role in the TME. On one side, TAMs possess the ability to absorb and eliminate debris originating from tumor cells, hence facilitating the proliferation and spread of tumor cells^12^. In contrast, TAMs were found to emit a considerable quantity of growth factors and cytokines, including vascular endothelial growth factor (VEGF) and tumor necrosis factor-alpha (TNF-α), to promote tumor angiogenesis and assist invasive tumor growth^13^.

As studies progressed, it became evident that TAMs possess anti-tumor characteristics^13^. The activation of TAMs was observed to induce alterations in their secretory profile, producing anti-tumor molecules that effectively suppressed tumor development and metastasis^14^. TAMs exhibit tight interactions with immune cells, hence influencing immunological responses, anti-inflammatory reactions, and the overall advancement of tumors^15^. It is noteworthy to acknowledge that the roles and phenotypes of TAMs exhibit a high degree of complexity, which is governed by multiple factors, including tumor type, cytokines present in the TME, and immunological signals^16^. Hence, comprehending and intervening in the mechanisms of TAMs can potentially facilitate the development of novel therapeutic approaches for combating tumors.

**Figure 1:**
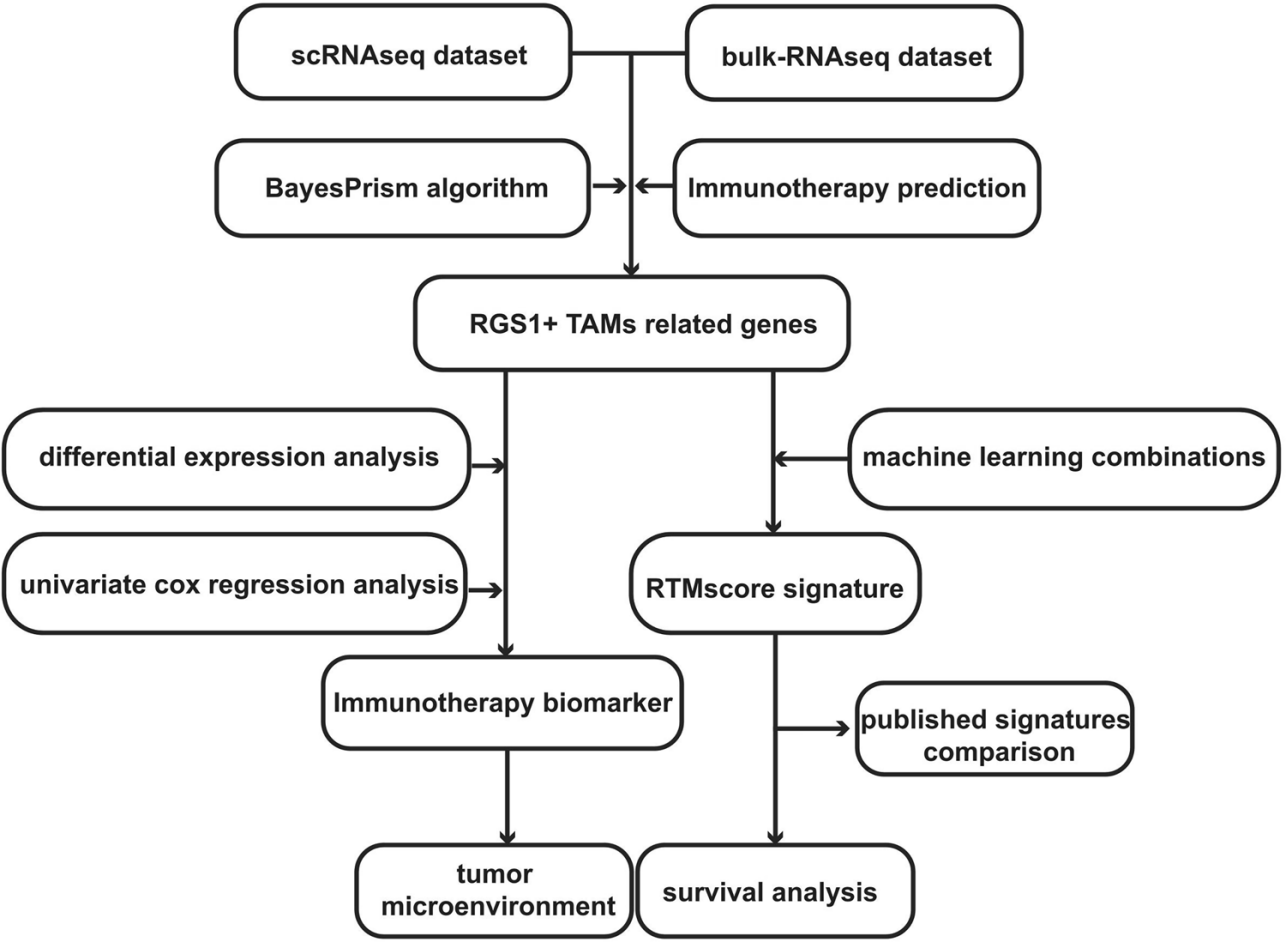
The study's flowchart diagram. Flow chart of the systematic identification of immunotherapy biomarker and the development of the RTMscore signature in LUAD. LUAD, lung adenocarcinoma; scRNAseq, single-cell mRNA sequencing; TAMs, tumor-associated macrophages; RTMscore, RGS1 + TAMs derived-genes score.

This study provided a comprehensive description of the immune cell composition in LUAD and characterized specific subpopulations of TAMs by analyzing single-cell mRNA sequencing (scRNA-seq) data. Additionally, the study made the novel finding of a previously unidentified subset of TAMs as RGS1 + TAMs that was strongly correlated with the therapeutic advantages of immunotherapy in patients with LUAD. The marker CD83 expressed by TAMs was identified as a means of distinguishing the appropriate population for immunotherapy. Furthermore, a predictive signature was developed to forecast the prognosis of patients. This was achieved through an analysis of the gene expression profile of RGS1 + TAMs and the use of diverse machine-learning techniques. The specific process of the study is shown in Fig. 1.

## Methods

### Data acquisition and processing

The study utilized normalized gene expression data and healthcare-related information, including healthcare status, disease diagnosis, treatment options, and medications, for a total of 493 patients diagnosed with LUAD. This data was obtained from The Cancer Genome Atlas (TCGA) (https://www.tcga.org) database^17^. The dataset from TCGA served as the training set for developing the RGS1 + TAMs derived genes score (RTMscore) signature. The criteria for selection were as follows: 1. The survival information of the patients was documented. 2. The individuals in question were diagnosed with LUAD. The GSE30219^18^, GSE3141^19^, GSE72094^20^, and GSE50081^21^ datasets, which consist of RNA-Seq data and complete clinical data (The definitive clinical features are listed in Supplementary Table 1), were acquired from the Gene Expression Omnibus (GEO) database (http://www.ncbi.nlm.nih.gov/geo/). These datasets were utilized as a validation cohort to evaluate the robustness and applicability of the RTMscore signature.

The researchers obtained single-cell RNA transcriptome data from patients with LUAD from the Gene Expression Omnibus (GEO) dataset GSE117570^22^.

The present study utilized immunotherapy datasets including treatment information and RNA expression data obtained from various reputable sources, including online databases and published studies. Specifically, the datasets employed in this investigation encompassed metastatic urothelial carcinoma (mUC) dataset (IMvigor210)^23^, metastatic gastric cancer (mGC) dataset (Kim2018)^24^, non-small cell lung cancer (NSCLC) dataset (GSE126044)^25^, chronic lymphocytic leukemia (CLL) dataset(GSE148476) and melanoma datasets (melanoma1.GSE91061^26^, melanoma2.GSE35640^27^, and melanoma3.GSE115821^28^) (Refer to Supplementary Table 2 for details). The platform documentation associated with the Bioconductor annotation program was utilized to annotate the GEO dataset. The TPM expression values were derived from the FPKM expression values. The process of converting Count expression values to Transcripts Per Million (TPM) expression values was performed using the “IOBR” R package.

### Single-cell RNA sequencing analysis

To ensure the utilization of scRNA-seq data of superior quality, it was imperative to subject it to processing and analysis using the “Seurat” R package, incorporating meticulous filtering techniques. Cells of low quality were eliminated from the dataset through the employment of specific quality criteria, including cell quantity, gene number, and the number of unique molecular identifiers (UMIs) detected. The inclusion criteria for genes in this study required their expression to be observed in a minimum of three individual cells. Cells that exhibited expression of fewer than 50 genes were excluded from the analysis. Moreover, cells that demonstrated expression of mitochondrial genes exceeding 5% were also excluded from the dataset.

The scRNA-seq data underwent normalization using the “NormalizeData” function. Subsequently, the data was transformed into Seurat objects, and the “FindVariableFeatures” tool was employed to identify the first 1500 highly variable genes. Subsequently, the “RunPCA” function from the “Seurat” R package was utilized to execute principal component analysis (PCA) to reduce the dimensionality of the scRNA-seq data, focusing on the top 1500 genes. The JackStraw analytic method was utilized to discover principal components (PCs) that were deemed important. From this analysis, the top 15 PCs were chosen for further investigation in cell clustering. The selection of these PCs was based on their ability to explain a substantial part of the variance.

The functions “FindNeighbors” and “FindClusters” inside the “Seurat” package were employed to conduct cell clustering analysis. The construction of the k-Nearest Neighbour Graph is based on the Euclidean distance calculated in Principal Component Analysis (PCA). The "FindNeighbors" function is utilized to identify the nearest neighbors for each element in the image. Next, the "RunTSNE" function is employed to execute t-distributed random neighbor embedding (t-SNE). The process of cell aggregation was successfully proven by the use of t-SNE-1 and t-SNE-2.

The primary objective of the initial cell annotation was to analyze and categorize the prevailing cell types inside the TME. These cell types were identified based on specific markers associated with each group. The dominating cell types detected were epithelial cells, characterized by the presence of markers such as EPCAM, CDH1, KRT7, and KRT19. Immune cells were also identified using markers such as PTPRC, CD68, and JCHAIN. Additionally, stromal cells were identified based on the presence of the PECAM1 marker.

In addition, immunological cells underwent processing for extraction and reaggregation, using the established Seurat standard protocol. The annotation of clusters was conducted using reference data from the Human Cell Atlas and was further refined through manual adjustment based on specific cell-specific biomarkers. These biomarkers included CD79A for B cells, CD1C and FCER1A for dendritic cells, CD69, LYZ, LGMN, CSF1R, and CD14 for macrophages, S100A12, FCN1, and S100A9 for monocytes, NKG7, KLRD1, and KLRB1 for NK cells, JCHAIN, IGKC, and IGHG1 for plasma cells, and CD3E, IL7R, CD40LG, CD8A, and CCL5 for T cells.

Then, tumor-derived macrophages were isolated and subsequently reaggregated using the “Seurat” R package. The reaggregated macrophages were then subjected to further analysis. The differential expression of genes among cell subpopulations was investigated using the “FindAllMarkers” function from the “Seurat” R package. The Wilcoxon test was employed to detect these differences. The “ClusterGVis” R package was utilized to conduct gene expression trends analysis and functional enrichment analysis.

### BayesPrism algorithm

BayesPrism algorithm utilizes RNA-seq samples from corresponding or comparable tissue types to unravel Bulk RNA-seq and spatial transcriptomics for cell type identification and gene expression analysis. It utilizes scRNA-seq data as prior knowledge to calculate the combined posterior distribution of cell type scores and cell type-specific gene expression within each batch.

BayesPrism algorithm comprises a deconvolution module and an integrated learning module. The deconvolution module creates a prior model using the cell type-specific expression profile of single-cell RNA sequencing data. It then estimates the cell type composition of RNA sequencing expression and the posterior distribution of cell type-specific gene expression for several tumor or non-tumor samples. The embedded learning module utilizes the expectation maximization (EM) technique to estimate tumor expression by combining malignant gene programs linearly. This estimation is based on the inferred expression level and the proportion of non-malignant cells calculated by the convolutional module.

### Collection of biomarkers in cancer immunotherapy

The relationship between the mRNA level of CD83 and immune cell infiltration was investigated based on the TIMER algorithm^29^, CIBERSORT algorithm^30^, quantiseq algorithm^31^, MCPcounter^32^, and EPIC algorithm^33^. Furthermore, the ImmuneScore, StromalScore, ESTIMATEScore, and Tumorpurity were determined from the analysis of distinct gene expression characteristics exhibited by immune and stromal cells, utilizing the ESTIMATE algorithm^34^.

In addition, a total of ten immunotherapeutic biomarkers were included in the study. The "easier" package^35^ was utilized for the computation of various immunological parameters, including Cytotoxic activity (CYT)^36^, IFNy signature (IFNy)^37^, Roh immune score (Roh_IS)^38^, chemokine signature (chemokines)^39^, Davoli immune signature (Davoli_IS)^40^, extended immune signature (Ayers_expIS)^37^, T cell-inflamed gene expression profile (GEP)^37^, immune resistance program (RIR)^41^, and tertiary lymphoid structure (TLS)^41^. TIDE scores were obtained from the TIDE website (http://tide.dfci.harvard.edu/)^42^.

### Integration of machine learning algorithms

To enhance the precision and consistency of the RTMscore signature, we incorporated ten machine-learning algorithms into our analysis. These algorithms encompass random survival forest (RSF)^43^, elastic network (Enet)^44^, Lasso^44^, Ridge^44^, Stepwise Cox^45^, CoxBoost^46^, partial least squares regression for Cox^47^, supervised principal components (SuperPC)^48^, generalized boosted regression modeling (GBM)^49^, and survival support vector machine (survival-SVM)^50^. Several algorithms have demonstrated the capability of doing feature selection, including Lasso, Stepwise Cox, CoxBoost, and RSF. Therefore, we integrated these algorithms to produce a consensus model. A total of 104 algorithm combinations were performed to construct prediction models using the ten-fold cross-validation technique.

### Validation and comparison of RTMscore signature

The research conducted an extensive review of existing literature about illness prediction models specifically connected to LUAD. The study then proceeded to compare the properties of the RTMscore with those of the published models to evaluate the predictive capabilities of the RTMscore signature. After excluding articles that did not provide clear prediction model formulas and articles that did not have matching gene expression data in the training and validation groups, a total of 102 prediction characteristics related to LUAD were selected. These characteristics include cuproptosis, ferroptosis, autophagy, aging, epithelial-mesenchymal transition, acetylation, amino acid metabolism, anoikis, DNA repair, fatty acid metabolism, hypoxia, inflammatory response, N6-methyladenosine, mitochondrial homeostasis, and mTOR, etc. (Reference to Supplementary Table 3 for more details) The scores were computed utilizing the algorithms outlined in the scholarly articles, and the C-indices of all prognostic indicators were calculated based on the log2-transformed TPM gene expression levels obtained from the TCGA database.

### Statistical analysis

The R package “DEseq2” was utilized to extract the mRNAs that exhibited differential expression between lung cancer samples and normal samples in the TCGA-LUAD dataset (log2FoldChange = 1.5 and padj = 0.05).

The prognostic value of the RTMscore signature was assessed by employing time-dependent receiver operating characteristic curves, utilizing the 'timeROC' R package. Statistical differences between groups for variables that follow a normal distribution were assessed using two-tailed t-tests. The Wilcoxon test was employed to ascertain statistical differences between groups for variables that were not normally distributed. The statistical analyses were conducted using the R program (version 4.1.2).

## Results

### Identification of the gene expression profiles associated with indicators of TAMs

**Figure 2:**
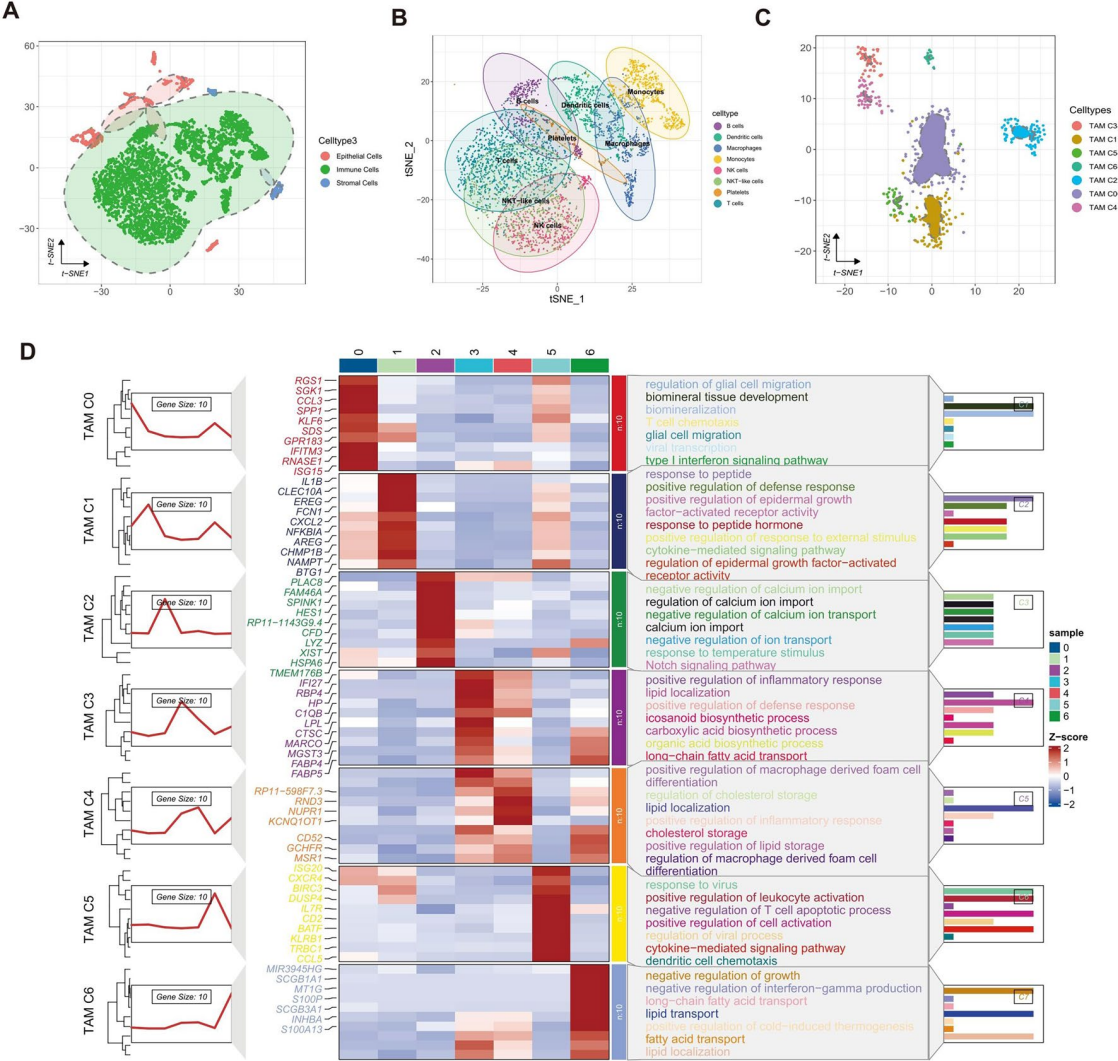
Deciphering of heterogeneity dominated by TAMs in LUAD. (**A**) t-SNE plot displaying the cell clusters in the microenvironment of LUAD. (**B**) t-SNE plot displaying the composition of immune cells in the microenvironment of LUAD. (**C**) t-SNE plot displaying the composition of TAMs in the microenvironment of LUAD, colored according to cell types. (**D**) The graph consists of three components. Left, lineplot displaying the expression trend of marker genes in different TAMs clusters. Middle, heatmap displaying the expression profiles of the top 10 genes ranked by LogFC of each TAMs cluster. Right, enriched GO terms for marker genes of each TAMs cluster.

The gene expression patterns of 5783 cells were analyzed in this study. The dataset used for analysis consisted of 4 LUAD samples and 4 normal samples of scRNA-seq dataset GSE131907. The data underwent filtration and extraction processes, resulting in the identification of 1500 variable genes. These genes were then used for reducing dimensionality and aggregating the 18 cell groups. The identification of immune, epithelial, and stromal cells was conducted using particular cell biomarkers (Figs. 2A and Supplementary Fig. 1A,B). This observation warrants further investigation.

The Human Primary Cell Atlas reference dataset was utilized to annotate a total of 4826 immune cells, along with the assessment of cell-specific markers' expression (Figs. 2B and Supplementary Fig. 1C,D). To further investigate the topic, 468 TAMs were isolated, given their significant role in the processes of carcinogenesis, development, and metastasis. A total of seven clusters were identified and subsequently labeled as follows: RGS1 + TAMs (TAM C0), IL1B + TAMs (TAM C1), PLAC8 + TAMs (TAM C2), IFI27 + TAMs (TAM C3), RP11-598F7.3 + TAMs (TAM C4), ISG20 + TAMs (TAM C5), and MIR3945HG + TAMs (TAM C6) (Fig. 2C,D).

### Immunological infiltration of RGS1 + TAMs significantly correlates with prognosis and immunotherapy benefit in LUAD patients

**Figure 3:**
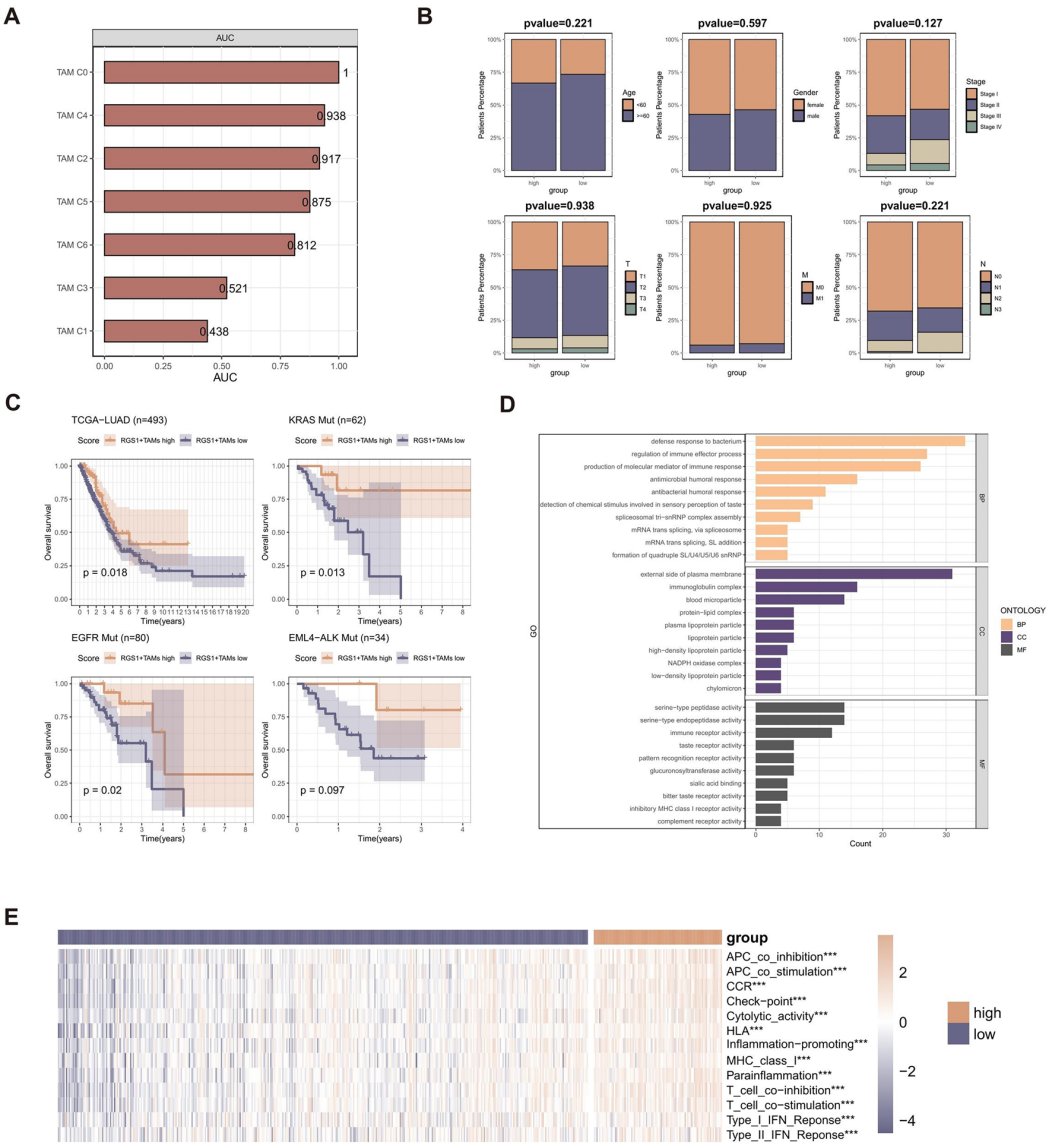
The relationship between the abundance of these RGS1 + TAMs and the clinical characteristics and immunotherapeutic characteristics. (**A**) Barplot displaying the area under ROC curves of the TAMs to predict the benefits of immunotherapy. (**B**) Comparison of proportions of subsets divided by age, gender, stage, T, N, and M between the group with high abundance of RGS1 + TAMs and low abundance of RGS1 + TAMs; the p-values were calculated based on the Wilcoxon rank-sum test. (**C**) Kaplan–Meier curves of OS according to the abundance of RGS1 + TAMs in the TCGA cohort (n = 493), EGFR mutation cohort (n = 80), KRAS mutation cohort (n = 62), and EML4-ALK mutation cohort (n = 34); the *p* values were calculated based on log-rank test. (**D**) GO enrichment analysis of the RGS1 + TAMs-related genes. MF, molecular function; CC, cellular component; BP, biological process (**E**) Heatmap displaying the correlation between the abundance of RGS1 + TAMs and 13 immune-associated processes.

The "BayesPrism" R package was utilized to ascertain the relative abundance of the 7 distinct types of TAMs present in both the GSE126044 and TCGA-LUAD cohort specimens. The study aimed to examine the correlation between the abundance of 7 distinct types of TAMs and the response to immunological treatment, which revealed a noteworthy association between the abundance of RGS1 + TAMs and the responsiveness of immune treatment (Fig. 3A).

To gain a clearer understanding of the correlation between the abundance of RGS1 + TAMs and other clinical variables, the study aimed to quantify the proportions of these variables in two subtypes of LUAD patients. The samples were categorized into two groups using the cutoff value of the abundance of RGS1 + TAMs obtained by the “survminer” R package. The results indicated that there was no notable association between the abundance of RGS1 + TAMs and clinical characteristics. (Fig. 3B).

To evaluate the potential differential function of RGS1 + TAMs in the presence of specific mutations, a Kaplan–Meier analysis was conducted to assess the survival disparities among patients with various gene mutations based on different abundance of RGS1 + TAMs. The results revealed that an increased abundance of RGS1 + TAMs in patients with EGFR mutations and KRAS mutations was associated with prolonged survival (Fig. 3C).

In light of the distinct prognoses observed in the two subtypes of LUAD, an analysis was conducted to identify differentially expressed genes (DEGs) that could elucidate significant variations in molecular function or components of the TME between these subtypes. The differential gene expression analysis was conducted to identify enriched Gene Ontology (GO) terms. The results revealed that the most significantly enriched terms were associated with immune-related functions, including immune receptor activity and inhibitory MHC class I receptor activity. These findings suggest distinct changes in the immunological ecology within the tumor between the two subtypes (Fig. 3D). The GSVA method was employed to compute scores for different immune-related functional signatures. LUAD patients with an increased abundance of RGS1 + TAMs demonstrated elevated immune functional scores compared to those with a lower abundance (Fig. 3E).

### The investigation of CD83's association with immunological features and its potential as an immunotherapeutic biomarker

**Figure 4:**
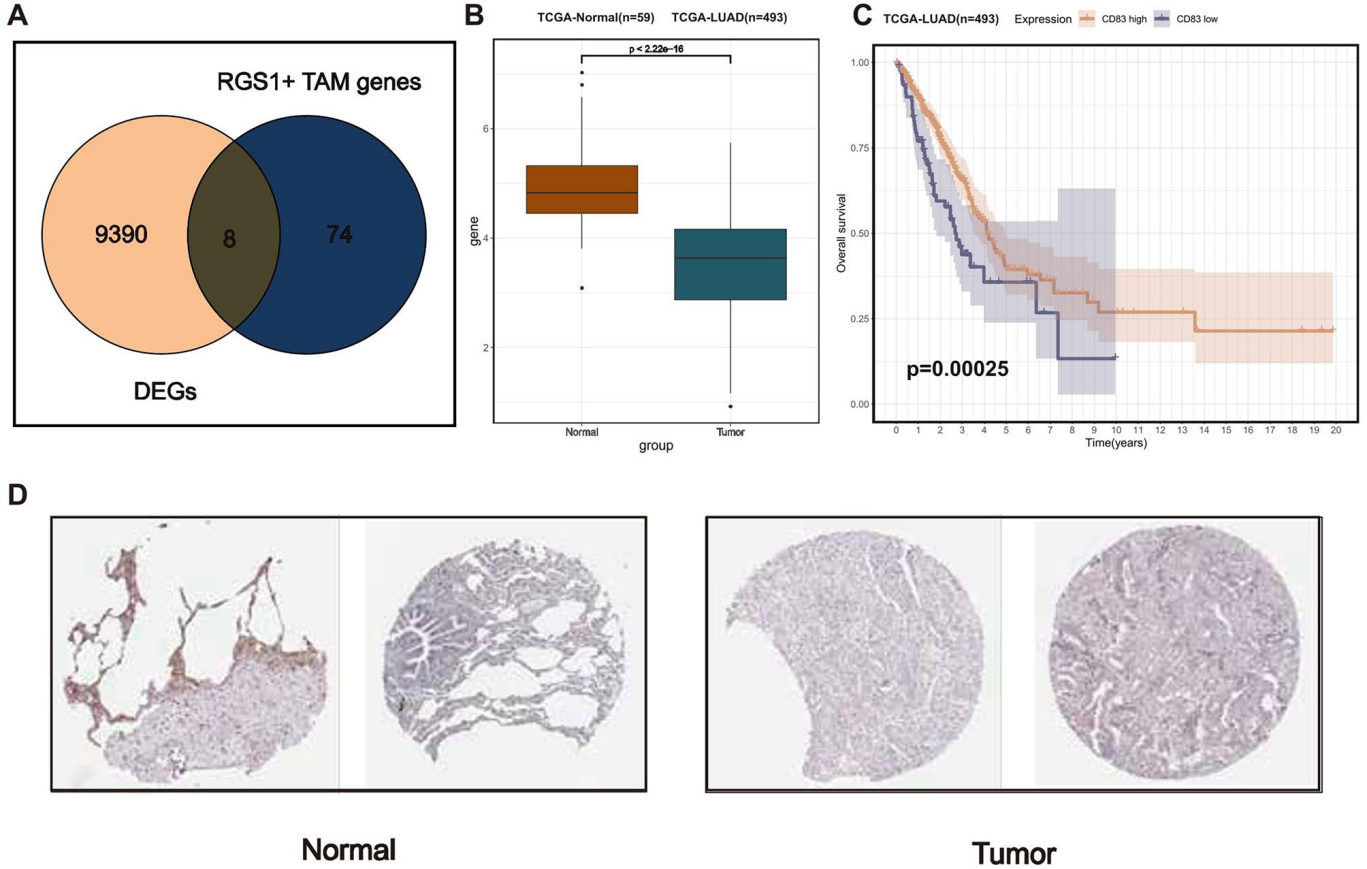
The identification of CD83. (**A**) Venn diagram representing the meeting point of RGS1 + marker genes and differently expressed genes between LUAD tissues and adjacent normal tissues in the TCGA cohort. (**B**) Different expression of CD83 between lung adenocarcinoma tissues and adjacent normal tissues in the TCGA cohort; the p-value was calculated based on the Wilcoxon rank-sum test. (**C**) Kaplan–Meier survival curve of OS between LUAD patients subjected to the high mRNA expression level of CD83 and the low mRNA expression level of CD83; the p-value was calculated based on the log-rank test. (**D**) Validation of the expression of CD83 in the normal and tumor samples level by the Human Protein Atlas database (immunohistochemistry).

The study conducted further analysis on the possibility of marker genes associated with RGS1 + TAMs as biomarkers for immunotherapy, given the strong correlation between RGS1 + TAMs and immunotherapy. With the intersection of the DEGs observed in lung cancer samples compared to normal samples, a total of 8 overlapping RGS1 + TAMs were extracted for subsequent analysis (Fig. 4A). Two genes, CD83 and A2M, were identified using a univariate regression analysis conducted on the hub genes (Supplementary Table 4).

Among these, CD83 had been previously identified as a distinctive marker for dendritic cells^51^. In recent years, a correlation was established between macrophages and CD83, which had been recognized as a novel immunological checkpoint in macrophages that played a role in the anti-inflammatory response^52^. Furthermore, it had been observed that CD83 had a role in the facilitation and expeditiousness of wound healing through the activation of macrophages that promoted wound healing^53^. The study aimed to further investigate the association between CD83 and LUAD. We investigated the clinical relevance of LUAD expression in patients who were diagnosed with LUAD. The Kaplan–Meier survival curves demonstrated favorable prognostic outcomes in LUAD patients who had increased levels of CD83 mRNA (Fig. 4B). The TCGA dataset was utilized to acquire data (Fig. 4C), which revealed a notable decrease in mRNA expression levels of CD83 in LUAD samples. The immunohistochemistry (IHC) analysis of the HPA dataset revealed a comparable dysregulation in the protein level of CD83 (Fig. 4D).

**Figure 5:**
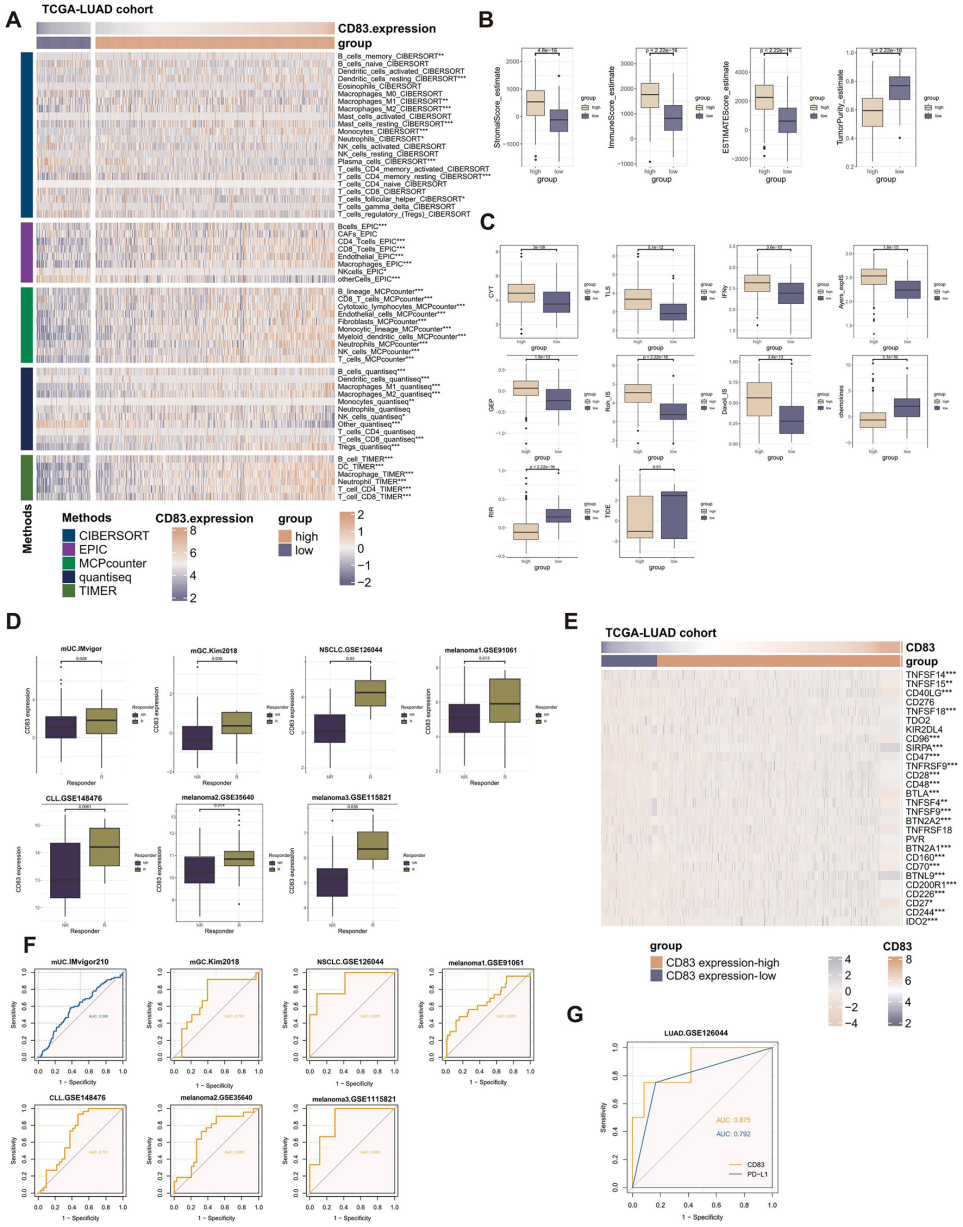
The CD83's potential as a biomarker for immunotherapy. (**A**) Heatmap displaying the correlation between the mRNA expression level of CD83 and immune infiltrating cells. (**B**) Box plot displaying the correlation between the mRNA expression level of CD83 and The ESTIMATE Immune Score, ImmuneScore, StromalScore, and TumorPurity. (**C**) Box plot displaying the correlation between the mRNA expression level of CD83 and immune modulators. (**D**) Box plot displaying the correlation between the mRNA expression level of CD83 and immunotherapy response in the immunotherapy cohorts (BLCA.IMvigor, STAD.Kim2018, LUAD.GSE126044, melanoma1.GSE91061, melanoma2.GSE148476, melanoma3.GSE35640, and melanoma4.GSE115821). (**E**) Heatmap displaying the correlation between the mRNA expression level of CD83 and immunological checkpoint genes. (**F**) ROC curves of the mRNA expression level of CD83 to predict the benefits of immunotherapy in the immunotherapy cohorts (BLCA.IMvigor, STAD.Kim2018, LUAD.GSE126044, melanoma1.GSE91061,melanoma2.GSE148476, melanoma3.GSE35640, and melanoma4.GSE115821). (**G**) ROC curves of the CD83 and PD-L1 to predict the benefits of immunotherapy. The p-values above were calculated based on the Wilcoxon rank-sum test.

The TME exhibited a significant association with the processes of cancer initiation, advancement, and the implementation of therapeutic strategies^54^. This study aimed to examine the correlation between CD83 and immunological characteristics to evaluate the involvement of CD83 in LUAD TME. A significant association was discovered between the expression level of CD83 and the extent of immune cell infiltration (Fig. 5A).

In addition, a statistically significant negative association was indicated between the expression of CD83 and the level of tumor purity (Fig. 5B). Moreover, a strong positive correlation was observed between the expression of CD83 and ImmuneScore, StromalScore, and ESTIMATEScore (Fig. 5B). The study also examined the association between CD83 and established immune modulators, including CYT, TLS, IFN, Davoli_IS, Roh_IS, GEP, and Ayers_expIS, as well as chemokines, RIR and TIDE (Fig. 5C). It was worth mentioning that the TCGA-LUAD cohort’s subgroup with a high expression level of CD83 exhibited significantly elevated levels of many factors, including CYT, TLS, IFN, GEP, Davoli_IS, Roh_IS, and Ayers_expIS, which were known to be associated with possible benefits of immunotherapy. The presence of low RIR, TIDE, and chemokine levels suggests a reduced probability of immunological escape.

The findings presented in this study suggest that CD83 has potential as an immunotherapeutic biomarker. Furthermore, it was observed that measuring CD83 mRNA levels may be more feasible compared to other immunotherapeutic biomarkers that require extensive experimentation and complex mathematical analyses but are seldom utilized in clinical settings.

Subsequently, given the strong correlation observed between the mRNA expression level of CD83 and tumor-infiltrating immune cells (TIICs), pathways involved in immunotherapeutic function, expression of immune checkpoints, and predictors of immunotherapy response, cohorts undergoing immunotherapy were included in the study to confirm the predictive significance of CD83 for the response to immunotherapy.

The responsive group for NSCLC patients in the NSCLC cohort had significantly higher levels of CD83 mRNA expression than the non-responsive group. The same trend was observed in the levels of CD83 mRNA expression of patients with chronic lymphocytic leukemia in the CLL cohort, patients with metastatic gastric cancer in the mGC cohort, and patients with metastatic urothelial carcinoma in the mUC cohort (Fig. 5D).

Meanwhile, in the cohorts melanoma1, melanoma2, and melanoma3, it was shown that the mRNA expression level of CD83 exhibited a statistically significant increase in the responsive group compared to the non-responsive group among melanoma patients (Fig. 5D). In addition, a notable correlation was observed between the mRNA expression level of CD83 and conventional immunological checkpoint markers (Fig. 5E).

The receiver operating characteristic (ROC) analysis conducted in the study showed that the mRNA level of CD83 exhibited a consistent ability to predict the efficacy of immunotherapy-based treatment. This finding was further supported by the analysis of gene expression datasets, including cohort mGC, NSCLC, melanoma1, CLL, melanoma2, and melanoma3, which yielded ROC values of 0.707, 0.875, 0.670, 0.701, 0.695, and 0.863, respectively (Fig. 5F). In terms of differentiating between Nivolumab responders and non-responders, the mRNA level of CD83 exhibited a substantially higher value (AUC = 0.792) compared to PDL1 (Fig. 5G).

### Construction and validation of the RTMscore signature based on the immune heterogeneity dominated by RGS1 + TAMs

**Figure 6:**
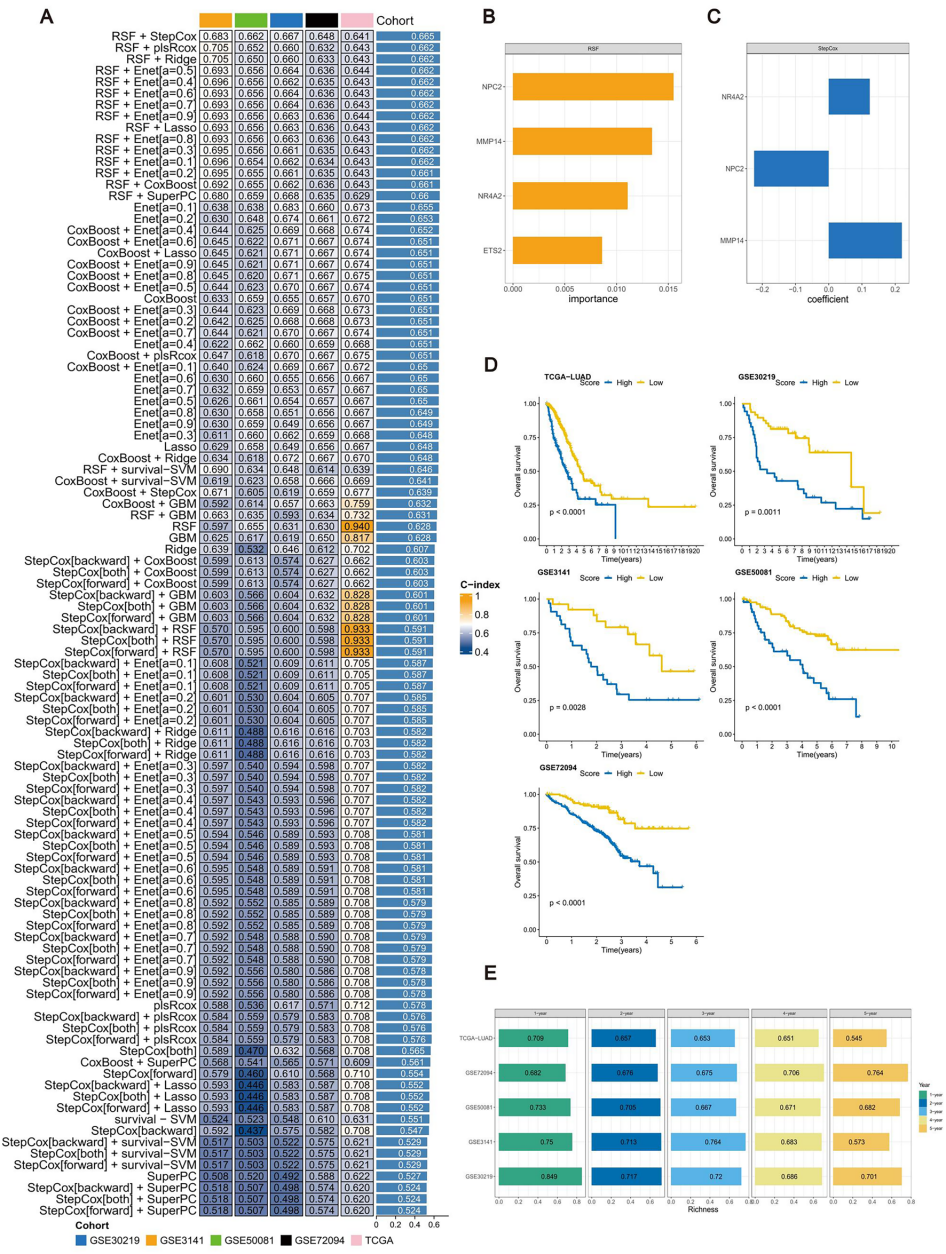
The construction and assessment of RTMscore signature. (**A**) A total of 104 combinations of machine learning algorithms for the RTMscore signatures via a tenfold cross-validation framework based on the TCGA-LUAD cohort (n = 493). The C-index of each model was calculated across validation datasets, including the GSE30219 (n = 83), GSE3141 (n = 58), GSE72094 (n = 393) and GSE50081 (n = 127) cohorts. (**B**) The importance of the 4 most valuable mRNAs based on the RSF algorithm. (**C**) The coefficients of 3 mRNAs were finally obtained in stepwise Cox regression. (**D**) Kaplan–Meier survival curve of OS between patients subjected to a high score of RTMscore signature and with a low score of RTMscore signature in TCGA-LUAD, GSE30219, GSE3141, GSE72094, and GSE50081 cohorts; the p-values were calculated based on log-rank test. (**E**) Time-dependent ROC analysis for predicting OS at 1, 2, 3, 4, and 5 years in TCGA-LUAD, GSE30219, GSE3141, GSE72094 and GSE50081 cohorts.

A total of 104 prediction signatures were derived by utilizing ten different machine learning methods, employing data from 82 RGS1 + TAMs-associated genes. The C-index was then computed for each of these signatures across all validation groups (Fig. 6A). The findings indicated that the RSF + StepCox approach demonstrated substantial predictive capabilities. This approach involved utilizing the RSF algorithm to identify four useful mRNA molecules (Fig. 6B). Subsequently, a Stepwise Cox proportional hazards regression analysis was conducted to choose three mRNA molecules (NR4A2, MMP14, and NPC2) as the final predictive signature (Fig. 6C). The equation that has been derived is as follows:

RTMscore = NR4A2 × (0.1235840) + MMP14 × (0.2199992) + NPC2 × (− 0.2247780).

The samples were categorized into groups using the cutoff value of RTMscore obtained by the "survminer" R package. The study conducted a Kaplan–Meier analysis to compare the high-RTMscore group with the low-RTMscore group (Fig. 6D). The results showed a substantial correlation between the RTMscore signature and OS in patients with LUAD from the TCGA-LUAD group. This association was further confirmed in independent cohorts, including GSE30219, GSE3141, GSE72094, and GSE50081.

The area under the receiver operating characteristic curve (AUC) values for the RTMscore signature were calculated for different time intervals (1-, 2-, 3-, 4-, and 5 years) in the TCGA-LUAD group. The estimated AUC values were 0.709, 0.657, 0.653, 0.651, and 0.545, respectively (Fig. 6E). These results indicate that the RTMscore signature has potential as a prediction tool for patients with LUAD. The validation of the model was performed in multiple cohorts, including GSE30219 (with AUC values of 0.849, 0.717, 0.72, 0.686, and 0.701), GSE3141 (with AUC values of 0.750, 0.713, 0.764, 0.683, and 0.573), GSE72094 (with AUC values of 0.733, 0.705, 0.667, 0.671, and 0.682), and GSE50081 (with AUC values of 0.682, 0.676, 0.675, 0.706, and 0.764).

**Figure 7:**
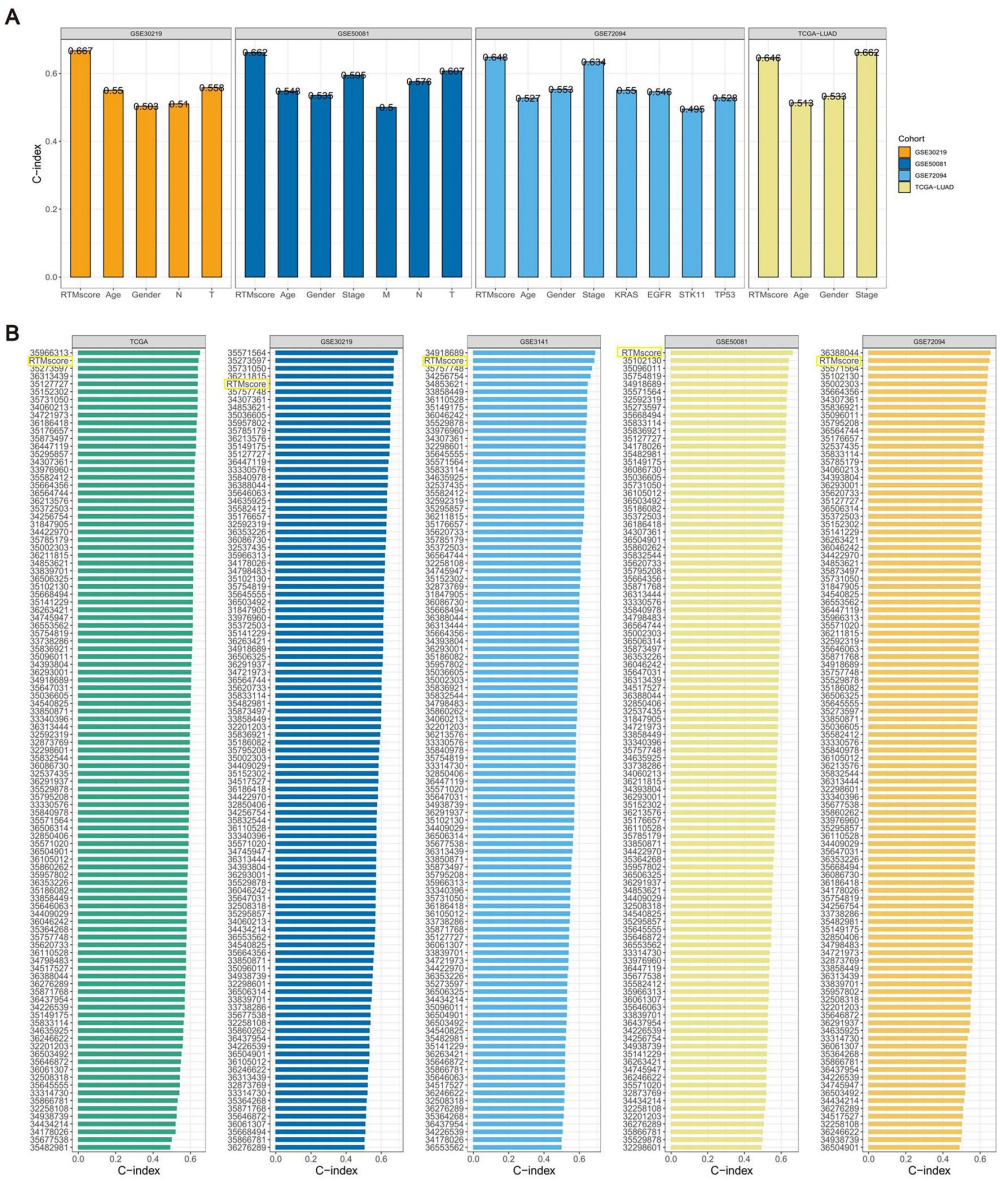
The comparison of the RTMscore signature with clinical characteristics and published signatures. (**A**) The C-index of the RTMscore signature and other clinical characteristics in the TCGA-LUAD, GSE30219, GSE3141, GSE72094, and GSE50081 cohorts. (**B**) The C-index of the RTMscore signature and other signatures developed in the TCGA-LUAD, GSE30219, GSE3141, GSE72094, and GSE50081 cohorts.

Furthermore, we conducted a comparison between the predictive value of the RTMscore signature and other clinical factors (Fig. 7A). The C-index of the RTMscore signature exhibited a much greater value compared to other clinical variables, encompassing staging, age, gender, and other relevant factors.

In recent years, predictive signatures have emerged significantly in machine learning-based gene expression analysis. This advancement has enabled the prediction of disease outcomes, facilitating early disease screening and the exploration of new therapeutic approaches. A literature search was conducted to compare the RTMscore signature with previously reported signatures in studies related to the LUAD-associated disease prediction model. After excluding articles that lacked explicit prediction model formulas and did not provide corresponding gene expression data in the training and validation groups, a total of 102 predictive signatures were linked with LUAD. These signatures were identified and validated using data from the TCGA-LUAD, GSE30219, GSE3141, GSE72094, and GSE50081 cohorts. The performance of these signatures was then compared to the C-index of RTMscore. The findings indicate that the RTMscore signature exhibited superior performance compared to the majority of signatures within its respective group, as seen by the results (Fig. 7B).

## Discussion

The treatment options for LUAD have become more diverse due to advancements in molecular biology and immunology, shown by the utilization of PD-1 inhibitors in conjunction with chemotherapy. The existence of a wide range of treatment options necessitates the development of more effective and individualized assessment procedures for patients to facilitate informed clinical decision-making. Nevertheless, a dearth of dependable prognostic indicators exists to identify individuals with “high-risk” LUAD who could potentially derive advantages from immunotherapy. To address this knowledge gap, we investigated the immune cell composition of LUAD and discerned distinct subpopulations of TAMs, specifically focusing on the characterization of RGS1 + TAMs. Our findings revealed disparities in the abundance of RGS1 + TAMs between the groups that exhibited a response to treatment and those that did not.

Furthermore, pathway analysis was conducted, revealing variations in the immune ecology within the tumor among the two subtypes. Additional examination revealed genes specifically expressed in RGS1 + TAMs. The association between the expression of the CD83 gene and the response to immunotherapy was observed among the subjects.

In addition, studies have shown that several types of immune cells, such as B cells, thymic epithelial cells (TEC), T cells, dendritic cells (DC), and neutrophils, have been identified to have CD83 expression. CD83 is crucial for activating T cells that control immunological responses in the body's periphery^55^. CD83 is a marker of mature dendritic cells (matDC) due to its increased expression during the maturation of dendritic cells^56^.

Data analysis based on clinical data also showed that patients exhibiting elevated levels of CD83 in the context of mUC, mGC, NSCLC, CLL, and melanoma demonstrated heightened susceptibility to immune checkpoint inhibitors. The immunomodulatory features of CD83 underscore its significant therapeutic potential.

In addition, the research effectively employed a total of 104 machine-learning algorithms to establish a robust RTMscore signature, which was created from genes associated with RGS1 + TAMs. The derivation of this signature was based on the analysis of many data sources, encompassing genetic markers, tumor characteristics, and macrophage-related aspects. The stability and durability of the RTMscore signature were ensured by leveraging the strengths of each algorithm and utilizing ensemble learning techniques. The utilization of the stable RTMscore signature has proven to be highly valuable in the domains of cancer research and precision medicine. The stability of the signature facilitated accurate prognostic predictions and enhanced comprehension of the intricate interplay between the TME and immune cells, hence leading to advancements in therapeutic approaches and eventually benefiting patient outcomes.

Furthermore, the RTMscore signature exhibited independent prognostic value, surpassing those of clinical parameters such as age, stage, and gender. In addition, it was observed that the RTMscore signature demonstrated a higher level of stability in its performance compared to a set of 102 previously published signatures when it came to predicting the prognosis. The majority of the signatures exhibited commendable performance on the training dataset; however, their performance on the validation datasets was subpar. This indicates a need for these signatures to possess greater generalizability. Simultaneously, the integration of different machine-learning techniques contributed to the robustness of our RTMscore signature.

The study undoubtedly has numerous limitations. Initially, validation of the RTMscore signature requires an independent clinical cohort, and additional data gathering is necessary to verify its predictive capacity. Besides, we integrated scRNAseq with bulk RNAseq data using the BayesPrism algorithm due to the scarcity of LUAD single-cell datasets on immunotherapy. Additional research is required to ascertain the function of RGS1 + TAMs in immunotherapy.

## Conclusion

In summary, the RTMscore signature introduced in this investigation exhibits promise as a prognostic indicator for patients undergoing treatment for LUAD, with implications for overall survival. Meanwhile, CD83 has the potential as an immunotherapeutic biomarker.

### Supplementary Information

Supplementary Figure 1.

Supplementary Tables.

## Data Availability

The analyzed data could be obtained from the TGCA database (https://portal.gdc.cancer.gov/), GEO database (http://www.ncbi.nlm.nih.gov/geo/), and TIDE database (http://tide.dfci.harvard.edu/). The code applied in the study is available from the corresponding author upon reasonable request.
