# Supplementary material for: Deciphering the heterogeneity dominated by tumor-associated macrophages for survival prognostication and prediction of immunotherapy response in lung adenocarcinoma
Source: Sci Rep. 2024 Apr 23;14:9276. doi: 10.1038/s41598-024-60132-4 (PMC11039664; doi:10.1038/s41598-024-60132-4)
Supplement: Supplementary file 1 — Supplementary Figure 1. [file 41598_2024_60132_MOESM1_ESM.pdf]

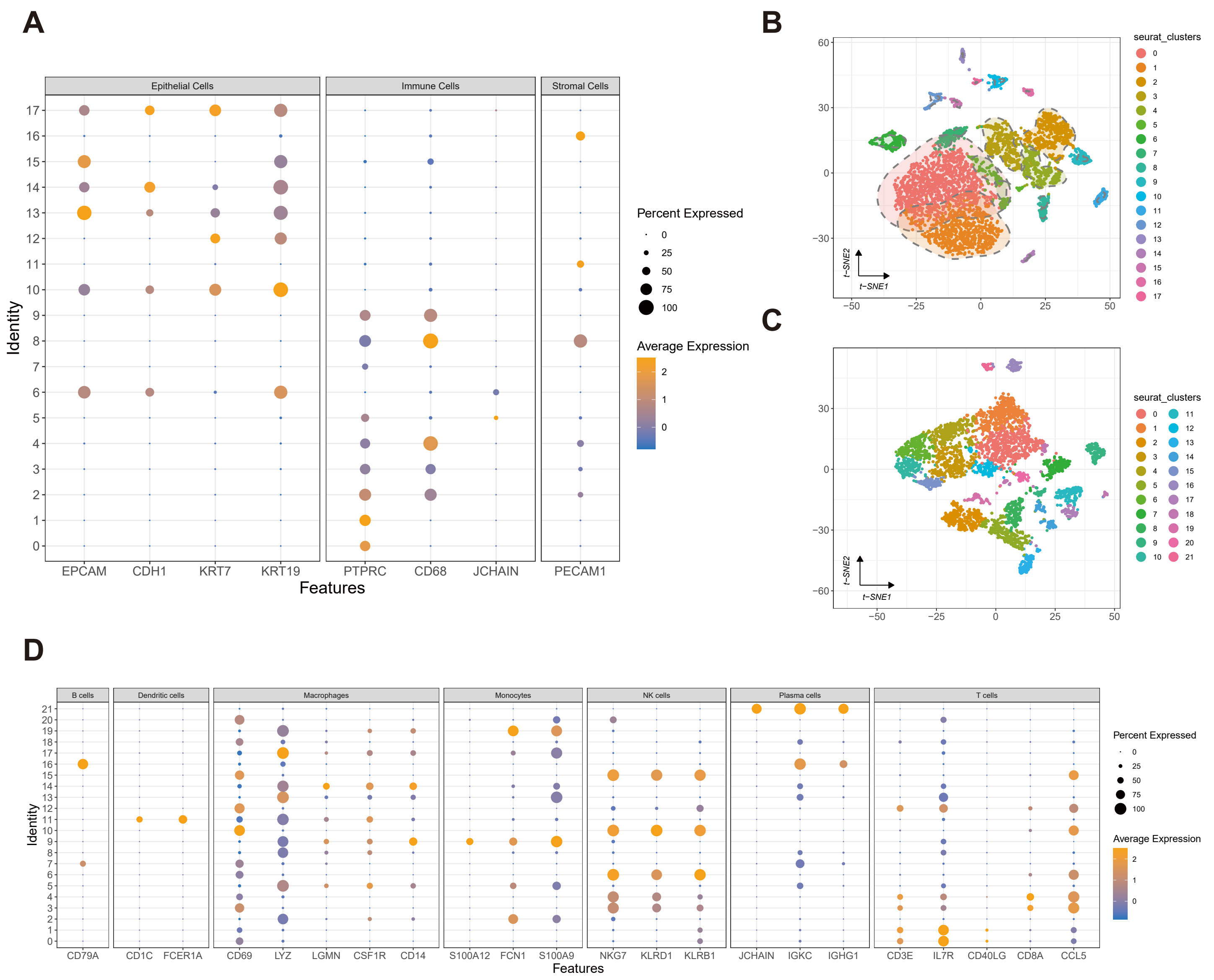

Supplementary Figure 1: The cell composition in the microenvironment of LUAD

(A) Dot plot displaying expression of cell-type markers across cell clusters. Dot size indicates the percentage of expressed cells, colored by their relative expression levels. (B) t-SNE plot displaying the cell clusters in the microenvironment of LUAD. (C) t-SNE plot displaying the immune cell clusters in the microenvironment of LUAD. (D) Dot plot displaying expression of immune cell-type markers across cell clusters. Dot size indicates the percentage of expressed cells, colored by their relative expression levels.
